# Vitiligo-like amelanotic melanoma in situ: A rare presentation

**DOI:** 10.1016/j.jdcr.2024.02.030

**Published:** 2024-03-16

**Authors:** Lilia Maria Lima de Oliveira, Uzma Farooq, Naiara Fraga Braghiroli

**Affiliations:** a Teaching Assistant at Harvard T.H Chan School of Public Health - Principles and Practice of Clinical Research (PPCR) - Post-graduate Program, ECPE, Boston, Massachusetts; b Dermatopathologist, Baptist Health South Florida, Miami, Florida; c Chief of Skin Cancer and Pigmented Lesions Clinic, Department of Dermatology, Miami Cancer Institute, Baptist Health South Florida, Miami, Florida

**Keywords:** achromic macule, amelanotic melanoma, amelanotic melanoma in situ, melanoma, melanoma in situ, skin cancer, vitiligo-like

## Introduction

Amelanotic melanoma (AM) is a rare subtype that accounts for 1% to 8% of all melanoma cases.^1^^,^^2^ AM tends to be more aggressive than pigmented melanomas and can be observed in all melanoma subtypes: superficial widespread, nodular, lentigo maligna, and acral lentiginous.^3^^,^^4^ AM is known as a masquerade tumor since it can mimic several dermatoses, commonly presenting as a nodule, erythematous scaly macule, or plaque with irregular borders. Thus, it takes a long time to be diagnosed, leading to a worse prognosis.^5^ We report a highly unusual case of a primary AM in situ, that presented as an achromic vitiligo-like macule with slow growth. This is the second such case published in the literature (Appendix 1).^6^

## Case report

**Fig 1:**
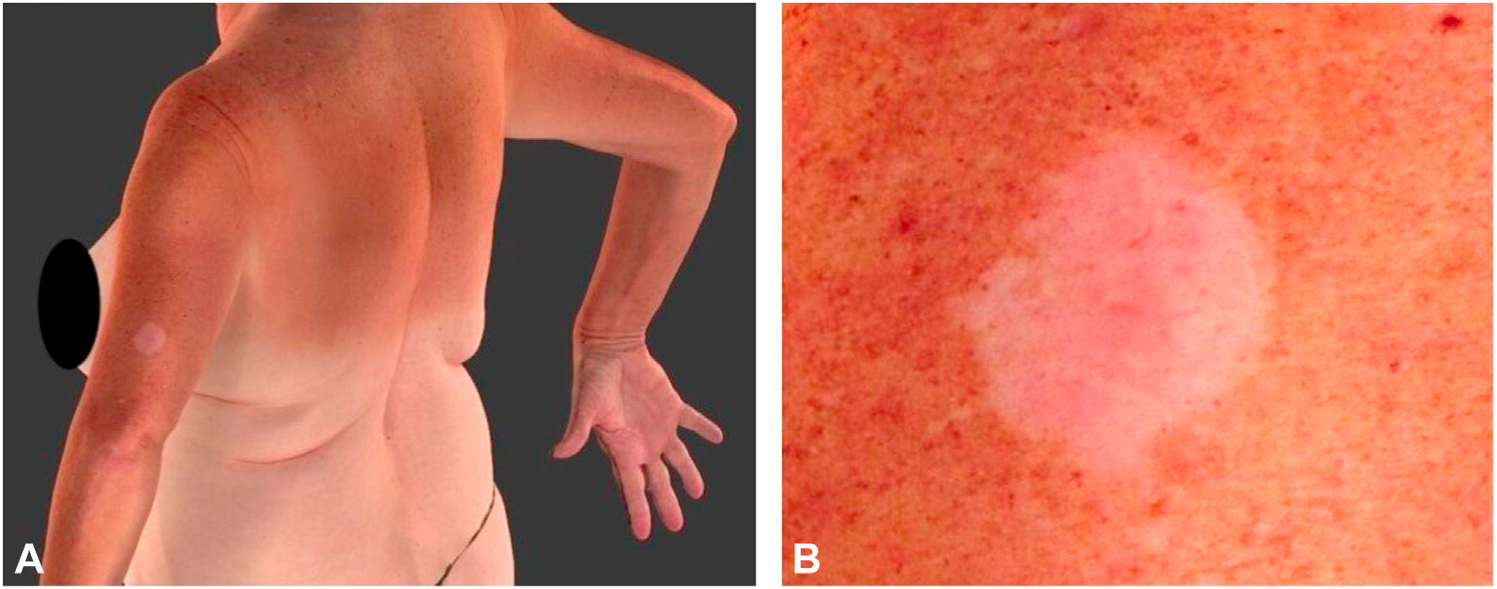
Clinical image, overview, and close-up, (**A** and **B**). A 2.8 × 2 cm, round, well-circumscribed, achromic, structureless patch on the left arm.

A 64-year-old woman, presented with a 2-year history of an asymptomatic white patch on her left arm. She denied a personal history of skin cancer, autoimmune disease, tobacco, and tanning bed use. The dermatological examination showed a 2.8 × 2 cm achromic, round, circumscribed, patch on the left arm. Telangiectatic vessels were evident in the amelanotic patch and adjacent areas (Fig 1, *A* and *B*). Significant sun-damaged skin was noted (Fig 1, *A*). No other similar macules or patches were present. A contact-polarized dermoscopy of the patch revealed a homogeneous achromic and structureless area.

**Fig 2:**
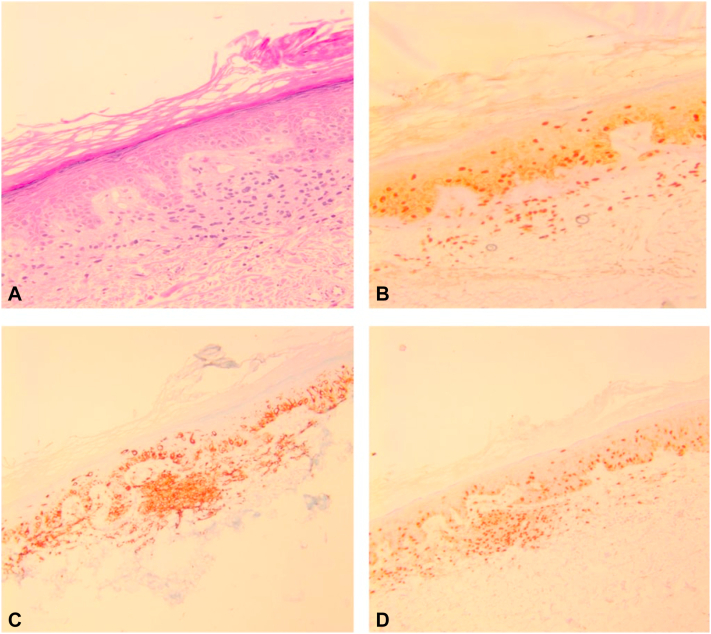
**A,** Histology (hematoxylin and eosin, 100×). There is a poorly circumscribed asymmetrical proliferation of melanocytes with severe cytologic atypia arranged in irregular nests and as single cells at the basal layer of the epidermis. A pagetoid scatter of melanocytes is identified. There is an underlying benign nest of melanocytes in the superficial dermis. Immediately bordering the melanoma in situ, the epidermis displays atypical melanocytic hyperplasia, which transitions to benign skin at the outer edges of the excision specimen. **B,** (Sox10, 200×) positive for atypical melanocytic cells, (**C**) (Melan-A, 100×) positive for atypical melanocytic cells, and (**D**) (microphthalmia-associated transcription factor, 200×) positive for atypical melanocytic cells.

The patch was treated as vitiligo with mometasone cream daily for 8 weeks. No repigmentation was evident 6 months after the initial visit. A biopsy was performed and histology revealed melanoma in situ with underlying dermal nevus remnant (Fig 2, *A*). Sox10, Melan-A, and microphthalmia-associated transcription factor stains were positive (Fig 2, *B-D* respectively). The melanoma was subsequently excised with 5-mm margins. The patient underwent 3-month follow-up examinations for 1 year, with no signs of recurrence. Currently, the patient remains under regular monitoring.

## Discussion

In the present case, we reported a rare case of vitiligo-like AM in situ, whose diagnosis was based on histopathological findings. There was no clinical or dermoscopic evidence supporting the diagnosis of AM. An isolated vitiligo-like macule and telangiectatic vessels that extended to the surrounding area were noticed throughout the dermatological examination. However, neither clinical nor dermoscopic evaluations revealed any specific vascular pattern, besides thin telangiectasias commonly seen on sun-damaged skin. Contact-polarized dermoscopy may explain why some telangiectasias were visible in the clinical examination but not under dermoscopic evaluation. The pressure on the skin caused by contact with the dermoscopy lenses compresses the vasculature and blanches the vessels. A noncontact dermoscopy was not performed.

Notably, the clinical diagnostic criteria for pigmented melanomas, such as asymmetry, irregular borders, color variation, diameter >6 mm, and evolution rule, do not apply to AM. It has been proposed that the EFG criteria (elevated, firm, growing for >1 month) be included in the asymmetry, irregular borders, color variation, diameter >6 mm, and evolution rule for the diagnosis of AM.^4^ However, the application of this criteria is limited because it can only detect nodular AM.^7^

Since clinical presentation is not consistently predictive of AM, dermoscopy can guide the diagnosis. The most specific morphological pattern and vascular distribution for AM are polymorphous vessels with a combination of 2 or more serpentine vessels, irregular linear vessels, dotted vessels, and hooked vessels.^4^

In addition, some factors, particularly if combined, can guide the clinician to consider an AM diagnosis when faced with a vitiligo-like macule, such as the following: (1) age over 50 years; (2) personal and family history of skin cancer; (3) use of artificial tanning; (4) fair skin; (5) sun-damaged skin; (6) presence of multiple moles; (7) isolated achromic or hypochromic lesion; (8); presence of atypical or polymorphous vessels on dermoscopy; (9) specific melanoma finds on dermoscopy; and (10) lack of improvement with standard vitiligo treatment. The presence of these features should raise suspicion of AM and therefore warrant a biopsy.

Primary AM in situ presenting as an achromic vitiligo-like macule or patch is extremely rare, with just 1 case reported in the literature.^6^ Given the rarity of the current case, the following clinical questions are raised: (a) Are AM melanomas underdiagnosed and underreported?^8^ (b) Is the prognosis of this type of AM better than classic AM? and (c) Could this be an undescribed subtype of AM?

This case report intends to increase clinical awareness and highlight the importance of considering AM as a differential diagnosis in patients with isolated slow-growing vitiligo-like macules unresponsive to standard treatment for vitiligo.

## Conflicts of interest

None disclosed.
